# Matrix of Architectural Solutions for the Conflict between Transport Infrastructures, Landscape and Urban Habitat along the Mediterranean Coastline: The Case of the Maresme Region in Barcelona, Spain

**DOI:** 10.3390/ijerph18189750

**Published:** 2021-09-16

**Authors:** Anna Martínez, Xavier Martín, Jordi Gordon

**Affiliations:** IAR Group, School of Architecture La Salle, Ramon Llull University, 08022 Barcelona, Spain; a.martinez@salle.url.edu (A.M.); jordi.gordon@salle.url.edu (J.G.)

**Keywords:** urban regeneration, littoral landscape, Mediterranean architecture, sustainable mobility, transport infrastructure, greenway

## Abstract

Maresme is a littoral region of Barcelona (Spain) in which the railway and an important road run along the coastline with a high landscape impact. Over time, several facilities connected to these transport infrastructures have appeared, such as industries, malls, marinas or train stations. These activities profit from the easy connection but create a barrier between the inhabitants and the sea. This research follows three aspects identified in a large variety of locations along the Mediterranean coast: longitudinal mobility, transversal accessibility and landscape discontinuities. The first territorial analysis defines a series of urban problematics classified by category. Then, the most representative case studies are developed by means of urban and architectural projects. The comparative analysis of these proposals provides a catalogue of design strategies which can be combined as criteria for solving multiple conflicts detected in the region. The result of this project is a methodology based on a matrix of general guidelines to ease the solving of local conflicts in a homogeneous way for the whole territory. The final aim is to re-establish order and continuity in the Mediterranean littoral skyline, fostering sustainable mobility and recovering public space for inhabitants.

## 1. Introduction

This research aims to provide architectural guidelines to solve mobility conflicts between transport infrastructures, landscapes and urban habitats, which are common in the seafront of the Mediterranean littoral [1]. Traditionally, free space between the sea and mountains has been a place for exchanges between civilizations and where the most intense activities have taken place [2]. For this reason, over time, it has been occupied by rural and fishing villages [3] as well as by industries and transport infrastructures [4].

Furthermore, the tourism boom has produced deep transformations in the economy and the way of living for these settlements [5], which have consequently increased accessibility conflicts around the sea due to urban sprawl and gentrification [6]. Recently, the maritime façade has once again become a busy place full of interactions between locals and foreigners, such as on cruise ships or leisure boats or through migrants [7]. In addition, civil society demands the use of the coast as an open public space, free and without physical or visual interruptions [8]. Finally, climate change drives us to rethink the way we occupy coastal territories and move through them in terms of space, time and energy consumption [9].

The present research is in the field of architecture, focusing on three particular action approaches: territory, building and constructive or climatic systems [10]. For this reason, common tools of architectural design are used to analyze, register, classify, synthesize, propose and show the methods. However, its purpose is not solving a professional architectural project but developing a research methodology in relation to an architectonic design process [11]. The practice of the project is systematized and settled from an abstract dimension, following architectural design methods based on synthesis and cause-effect relations [12].

Within this scope, the Maresme coastal region (Barcelona, Spain) is presented as a representative case study because of its conflicts between landscape and mobility (Section 2). This region consists of unique geography located between the mountains and the sea in a narrow piece of land occupied by a sequence of compact urban settlements, tourism sprawl, industrial parks and agricultural fields. In addition, due to the proximity of Barcelona, two large and intensive transport infrastructures have emerged along the seashore: the railway and the National II road (N-II). Due to their linearity, access to the beach and maritime landscape from coastal villages is almost blocked, affecting citizens and tourists. Therefore, the final purpose of this work is to foster more sustainable and healthier mobility systems (e.g., cycling, walking and swimming) [13] not only at a local level but also in relation to the capital city of Barcelona [14].

This research can be understood as an opportunity to solve these problems in a coherent way with the littoral landscape not only in one specific location, but also in other similar places all along the Mediterranean coastline following the methods presented [15,16,17,18,19,20,21]. Furthermore, in these littoral areas, there is a common conflict between the management of the different implicated administrations [22] (e.g., municipalities, provinces and government) and also between different infrastructures (e.g., railways, roads and ports). Hence, this research plans to establish unified criteria for the whole littoral that are able to solve most of the detected problematics. As a main result, it provides a tool to facilitate the application of solutions by different stakeholders with similar principles: the matrix of standardized design solutions.

This article shows the cartographic analysis (Section 3.1.), the development of the case study of the Maresme region (Section 4.1.) and the set of architectural guidelines defined as a matrix and the schemes of the standard solutions (Section 5). The visual and universal characteristics of these proposals generate a common base of understanding which aims to promote public debate about the aforementioned conflicts. Furthermore, this is the first step in the process to re-establish the order and continuity of the seafront [23], enhancing healthy mobility and recovering the accessibility of public spaces to citizens [24].

## 2. Hypothesis, Objectives and Case Study: The Maritime and Urban Landscapes of the Maresme Region

This research is focused on answering the following questions. First, how can architectural designs contribute to generating more sustainable mobility and reducing motor transport intensity, such as by train or car? Secondly, which public space design patterns are useful to provide free, open and high-quality landscape accessibility to inhabitants? Finally, is it possible to generate a system of solutions with unified criteria to add order and dignity to the maritime facade based on solving issues in different locations with similar casuistry at a local scale instead of a unique larger project?

The aim of this work is to offer regeneration strategies for mobility and landscape conflicts, understood as guidelines which can be used for different administrations at different scales and with different deadlines but with unified criteria [25]. The following list notes the most representative objectives that set the scope of this research:

- Freedom of movement: the rationalization of mobility and accessibility, ensuring the continuity of pedestrian routes and road paths; pacification of circulation systems; promotion of sustainable and healthy mobility (cycles and pedestrians) and introduction of new types of transport to diversify and unload its intensity [26].
- Sight continuity: the consideration of long-distance views between urban and coastal landscapes; preservation of the continuity of the horizon from inland and definition of the maritime façade and its appearance from the sea [27].
- Compatibility of uses: strengthening the heterogenous character of the littoral; promotion of economic diversity considering existing uses and setting their physical and temporal combination with the new ones and transformation of obsolete buildings and areas with potential for urban regeneration [28].
- Character of place: preservation of historic and natural sites, namely those important for the memory of the place; identification of traditional characteristics related to urban and agricultural structures and the introduction of these activities to the new dynamics of the territory [29].
- Mediterranean culture: the reinforcement of traditional sustainability criteria used by local cultures and following their ways of living and the adoption of the Mediterranean passive systems and materials, including vegetation, to face climate conditions in the design of buildings and public spaces [30].

**Figure 1:**
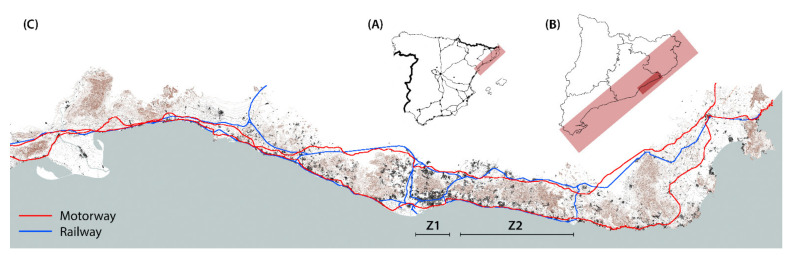
Index map of the Catalan coast and location of the Maresme region (Z2) in relation to Barcelona (Z1) (Source: authors’ own). (**A**) Location of the Catalan coast in Spain. (**B**) Location of the Maresme region on the Catalan coast. (**C**) Index map detail: topography, hydrography, urban settlements and transport infrastructures.

The research methodology is applied to the case study of Maresme. This region extends along the northern littoral of Barcelona in a narrow territory between the littoral mountain range and the Mediterranean Sea [31]. Its coast length is about 50 km, and the width between the sea and the mountains varies between 5 and 15 km [32] (Figure 1).

Traditionally, in this region, transport infrastructures have been placed parallel and close to the coastline, following its uniform topographic level [33]. The first railway built in Spain has connected Barcelona and Mataró (capital of Maresme) since 1848 [34], and the N-II road connects Madrid and France through Barcelona [35]. Both infrastructures have undergone different transformations (e.g., extensions, bridges or roundabouts), but the route along the seafront has been maintained. Coastal villages are located all along the road and connected inland following streams and riverbeds, which are the most representative landscape elements in Maresme [36].

Maresme is a territory with a deep agricultural and industrial tradition [37], although nowadays it is oriented to the third sector, including services and tourism [38]. From the mid-20th century, the expansion of leisure activities fostered the occupation of this territory by means of secondary residences, which finally developed into a stable population [39]. Villages have extended inland and over the hillsides in an urban sprawl configuration. This situation implies an important increase in daily traffic between Maresme’s villages and the city of Barcelona, both by train and by car [40].

**Figure 2:**
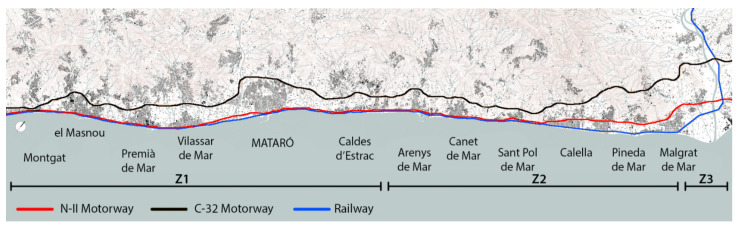
Transport infrastructures follow the coastline of Maresme and relate three landscape units: Baix Maresme (Z1), Alt Maresme (Z2) and Baixa Tordera (Z3) (Source: authors’ own).

In relation to the sea, its coastline is a continuous facade that has been compacted by the systematic construction of industrial parks and public facilities in both margins of the urban settlements. Despite this repeated pattern on the littoral front, the region is divided into three landscape units which define their particular values in relation to identity, geography and activities: Baix Maresme, Alt Maresme and Baixa Tordera [41]. The geographic range of this research starts in Montgat and ends in Malgrat de Mar, where the railway goes inland. A series of similar landscape elements link 10 municipalities located along the seafront (Figure 2). It is a region practically built in all its length, and rural spaces between the urban settlements are mainly occupied by crops and campsites [42].

**Figure 3:**
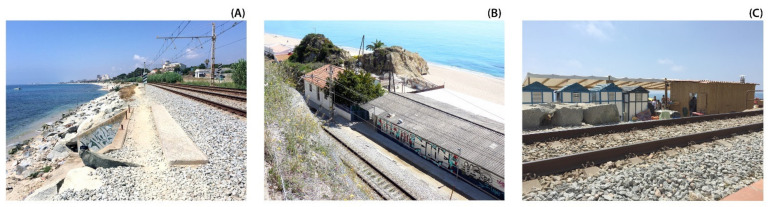
Fieldwork views of the Maresme littoral (Source: authors’ own). (**A**) Canalized stream mouth below the railway. (**B**) Factory located between the railway and beach. (**C**) Beach bar and facilities close to the railway.

At the edge of this fringe, the N-II road and the railway run longitudinally and in continuity with the coastline. In addition, some cycling lanes and pathways follow this route, but they are fragmented and have several topographic and physical obstacles [43]. This grouping of infrastructures has an average width of about 40 m, but in some specific spots it can be 180 m, with significant unevenness between the interior side and the seaside. Thus, transversal accessibility from the inhabited area toward the sea is irregular due to the multiple types of obstacles and usually narrow public spaces of low comfort quality (Figure 3A). All along this region, due to its particular orography, the presence of riverbanks and streams which flow into the beach is also common, sometimes with significant floods [44]. These hydrographic elements successively cross underneath the infrastructures and are commonly used by citizens to reach the sea [45].

Finally, the seafront has been traditionally occupied by industries due to the easy connectivity by both land and sea (Figure 3B). However, nowadays, very few are still working, and they have instead been replaced by modern industrial parks in the urban peripheries. These historic factory buildings are part of the culture of the site, and even though some of them have been demolished, the most representative ones are being refurbished or are due to be repurposed for new public uses (e.g., the Anis del Mono factory in the nearest coast of Badalona or the flour factory Ylla-Aliberch in Mataró) [46].

Recently, huge commercial malls have occupied areas of this coastal fringe, mainly in the boundaries of the villages. These spots also comprise transport nodes, roundabouts and parking lodgments to provide access to public services and allow inhabitants to access the beach. Both typologies use an important amount of land, despite being located in a privileged landscape facing the sea. Furthermore, the successive presence of nautical clubs and marinas creates restrictions on the freedom of movement and continuous circulation of inhabitants in relation to the beach (Figure 3C).

Finally, regarding climate change, in the Catalan coast, there is a common meteorological phenomenon which produces strong storms from the sea toward the land (llevantades, related to the levant wind). This rough weather drags sand and destroys beaches and seafronts, flooding constructions located along the coast. The latest notorious example is Storm Gloria in January 2020 [47].

## 3. Methodology

### 3.1. Mediterranean Strategies Methodology

This research project followed the methodology established in the research line “Mediterranean Strategies” of the Integrated Architectural Research (IAR) Group, applied by the authors in previous similar studies [48]. This was developed in an inductive process which sought to obtain new ways of acting from the specific study and resolve some representative cases, which were chosen after a deep analysis of the site. The aim was to set a series of guidelines that could be applied to all the identified cases along the territory and also in other similar regions or locations. This investigation focuses on the architectural design, based on the graphic registry as a tool for representation and idea development [49].

Thus, the research methodology was based on the stages noted in Table 1.

#### 3.1.1. Analysis and Classification of Problematics

**Figure 4:**
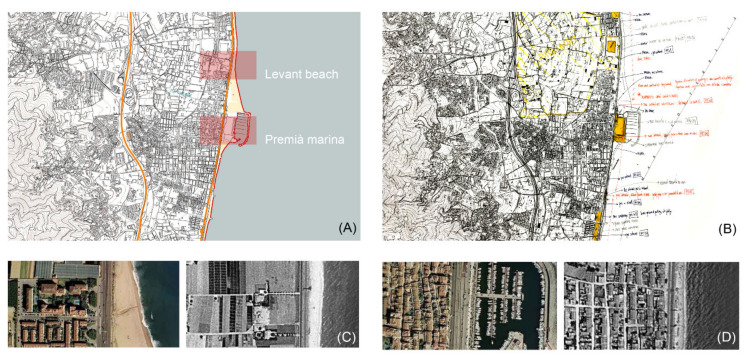
Cartographic analysis based on fieldwork and archive mining (Source: authors’ own). (**A**) Digital mapping of the Premià de Mar (Sea Premià) area. (**B**) Fieldwork draft map of Premià de Mar. (**C**,**D**) Sample of diachronic analysis by comparison between the current aerial view and the view from an American flight (1956) [51].

The first step in the research was the cartographic analysis of the territory (Figure 4). For this purpose, current situation maps were drawn based on fieldwork and on digitalization of existing cartographies [50,51]. Visits to the site were recurrent in order to validate the online information. Drawings and schemes were developed following a common graphic criterion, based on the selection of collected information and in relation to the expected use of these mapping documents (e.g., project requirements, analytical phases, scales and formats) [52].

At the same time, other intangible elements of the territory were analyzed in order to determine the causes of its current degradation: the history, economy, traditions and culture of the place. The combination of these two approaches on the site eased the detection of its different problematics, which were identified and classified by typologies. This phase ended with the selection of representative study cases [53].

#### 3.1.2. Regeneration Projects

Based on the classified problematics, some study cases were selected as a representative sample of the typological conflicts to be solved. Working on a specific study case instead of a whole territory provided better defined and detailed solutions [54]. In addition, developing specific proposals from urban and architectural processes provided solutions that were more related to the context. Despite the use of architectural design tools, the proposed solutions have an abstract character, since they are based on universal elements [55]. They are presented with visual drawings and models to be comprehensible for all stakeholders.

The proposals of these particular projects are intended to be developed from the strict conditions of the place and paying attention to urban parameters and current constructive systems. With these criteria, new uses and facilities can be proposed with no alteration of their presence in the urban landscape while fostering sustainable solutions in relation to the Mediterranean weather conditions [56].

#### 3.1.3. Intervention Strategies

Finally, the different solutions proposed for each case study are compared and synthesized as action guidelines. These strategies recognize the site integration criteria, the project definition, constructive execution and climate protection. These actions can be applied to all different locations within the territory in a unified sense but are flexible enough to be adapted to each site [57]. The final result of the research is not an architectonic project but a series of basic guidelines to be developed in each of the locations by different professionals and administrations. The methodological process allows for defining these universal criteria and aims to establish strategies to reinforce the quality of the littoral landscape and foster its public use for future generations [58].

### 3.2. Research Lines’ Definitions

The first territorial analysis of the Maresme case study highlighted certain problematics that are repeated all along the coast. The study was organized into three research lines related to three different approaches to the territory: longitudinal continuity, transversal permeability and urban landscape. Each line was defined by specific objectives in relation to the identified problematics, mobility and the geographic context. The multiple combinations of these three approaches and their solutions will create an adaptative system to solve all the different conflicts detected by means of unified criteria.

#### 3.2.1. Longitudinal Continuity

**Figure 5:**
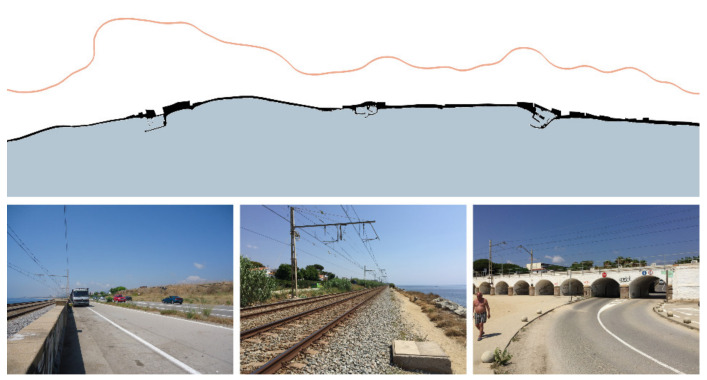
Longitudinal continuity studies the effects of the infrastructures and the civic ways which connect different urban settlements all along the coast (Source: authors’ own).

Infrastructures and facilities are usually connected following a route parallel to the coastline (Figure 5). However, there are relevant differences between the main roads of fast mobility (car, train or bus) and secondary pathways of slow and light mobility (pedestrians or bicycles). There is also a distinction between long-distance trips, mainly related to Barcelona, and the short-distance ones between villages within Maresme.

Railways run along a uniform topographic level, usually delimited by fences on both sides. Depending on the area, this infrastructure consists of one or two lines in both directions. Every village has a station or a stop, and sometimes even two of them. Most of the platforms have been lengthened to support longer trains, as the frequency of services and volume of users are very high during rush hours [59]. Platforms are usually accessed by underground corridors. Nowadays, ticket validation is automated, so most of the traditional buildings have been transformed or closed to the public.

Despite being situated at the seafront, it is normal that in the short term, there is not a clear plan to get rid of this type of railway infrastructure [60]. In the city of Mataró, there is an urban project to modify its route inland, but it is currently unaffordable in terms of costs and political management [61]. In any case, there is a plan to transform this railway into a tram that is slower and less aggressive in relation to the landscape [62]. Therefore, the case study projects take the following consideration into account: keeping the same route but with a lower traffic intensity.

The N-II road has never had its route modified within the Maresme region. It connects all villages in the area and extends toward Barcelona to the south and toward Girona and France to the north. This road has two-way traffic, with three to four lanes in urban areas and up to six lanes in some inter-urban zones. Inland connections and village access are solved with roundabouts and crossroads. Here, there is a plan for directing part of the traffic supported by the N-II road to other existing highways inland, such as the Comarcal 32 (C-32) motorway, which is faster and wider [63]. This way, in the future, this road will be converted to a civic pathway, adequate for local or regional traffic. This future condition is also taken into account in the case study proposals.

Both infrastructures (railway and N-II road) are continuous elements with a strong presence, sometimes even located at different topographic levels. On the contrary, pedestrian and cycle pathways along the seaside are discontinuous and fragmented but with great potential. These pathways are recognized as part of the history of the region, being very attractive in terms of landscape and ability to foster sustainable and healthy mobility [64]. These routes are commonly located in the narrow space between the railway and the sea. Obstacles might be produced by natural elements (e.g., streams or topography), by buildings or facilities (e.g., stations, marinas), by infrastructure (e.g., underground passages) and also by different urban treatments in the boundaries between municipalities.

This research line recognizes the necessity of giving continuity to these relatively small and healthy longitudinal itineraries, which are mostly used by the inhabitants in short trips or even related to leisure and tourism activities. However, these could also support longer distances and higher volumes if they became easy to use, comfortable and secure. The design and measures of these external lanes have to be developed considering the storms coming from the sea and the water level rising [65].

The specific objectives of this research line consist of pacifying and diversifying the different types of traffic and ensuring the coherence of their routes parallel to the coastline. For this purpose, two parallel and continuous ways can be disposed of in relation to the central railway: a civic pathway on the inner side and a landscape-friendly greenway next to the sea on the outer one.

#### 3.2.2. Transversal Permeability

**Figure 6:**
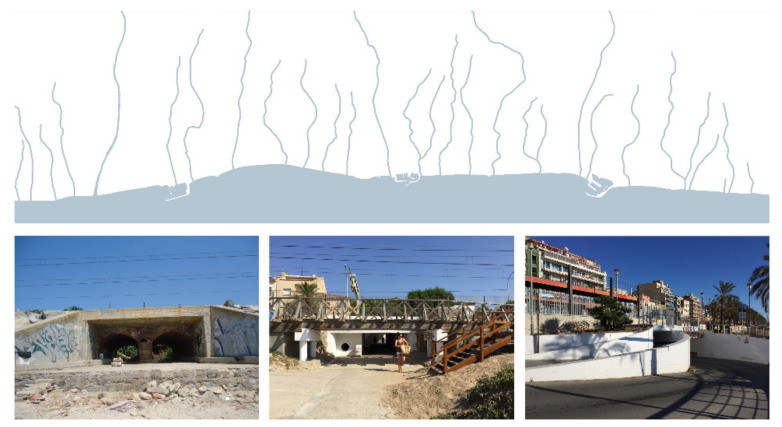
Transversal permeability studies the connectivity between the inland area and the beach, following the streams which cross infrastructures through underground passages (Source: authors’ own).

Maresme’s hydrographic river basin is singular due to the fast overflow of rainwater and conflicts generated by the inefficiency of the stream mouths, which need to cross below coastline infrastructures toward the sea [66]. This problem is similar for the inhabitants and tourists’ mobility from villages facing the beach. They also have to cross the coastline infrastructures, which are sometimes highly uneven, implying an unacceptable lack of safety (Figure 6).

This transversal connectivity for water, vehicles and pedestrians is solved in many different ways all along the coastline but usually with no order and without any comfort quality. Some streams have already been canalized, but most of them still flow into the beach, as do some sewer pipes and local overflow channels. Furthermore, access for vehicles to the coast is made possible through underground passages connected to the urban road system. There are few of these in relation to pedestrian ones, and the conditions for construction (e.g., measures, slope, curves and lane width) usually require a large amount of land to be occupied with no other use. Furthermore, these vehicle passages do not take into account compatibility with pedestrian circulations.

Finally, the majority of pedestrian access routes to the beach are underground passages. These can be public or private and are regularly distributed along the coast. Most of them are former sewer pipes, so they do not meet the minimal measure requirements nor do they provide comfort or safety. Sometimes, railway platforms are also used to cross the infrastructure, and there are very few examples of elevated footbridges.

The main objective of this second research line is to ensure transversal accessibility to the coast from inland urban settlements, rationalizing their location and enhancing the quality of underground passages. The plan is to set a regular pattern of passages along the coast in both urban and inter-urban areas equally and especially in the crossroads between longitudinal infrastructures and transversal axes (e.g., streets and pathways). It is also important to provide spatial and comfort quality for these passages, fostering their use as a public space and an agent of dynamic urban activity between both sides of the railway. It will be necessary to include design solutions to keep mobility safe even when floods happen. Solutions for extreme flooding and natural disasters are not part of the aims of this project.

#### 3.2.3. Urban Landscape

**Figure 7:**
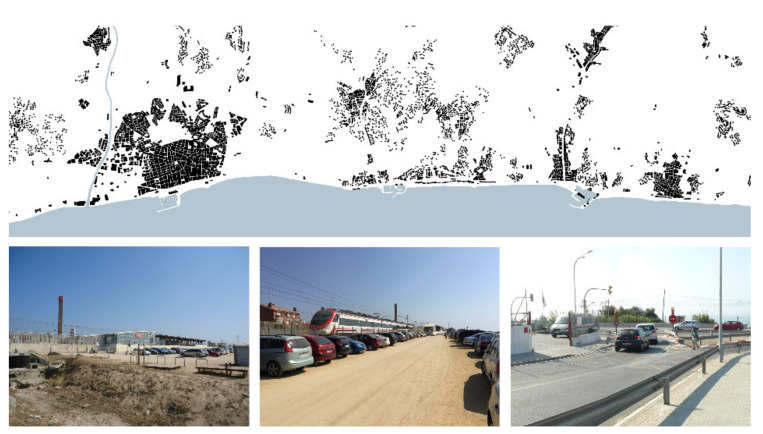
Urban landscape studies obsolete enclosures and buildings with multiple typologies, which become discontinuities in a general view of the littoral landscape (Source: authors’ own).

The case studies identified in this research line refer to isolated or extensive constructions of different sizes and also to urban voids which produce important discontinuities in the seafront (Figure 7). These are equally located on both sides of the infrastructures in urban and inter-urban areas. Some examples of these constructions are disused buildings, those undergoing a transformation process (e.g., factories and railway stations), enclosures of private complexes (e.g., nautical clubs, marinas and campsites) and parking lodgments related to the beach, commercial areas and stations.

Regeneration of obsolete buildings as well as the definition of links between urban voids and natural free spaces provide better landscape conditions [67]. Due to their capacity to support new uses if needed, these areas of conflict might become nodes of activity and provide a great opportunity to organize longitudinal and transversal circulations [68].

This research line aims to recover the quality and continuity of the seafront by means of balancing the presence of infrastructure, buildings and open spaces. Projects will enhance the value of the littoral landscape, considering all the elements which are part of this [69] (i.e., not only the physical ones, but also visual, cultural, urban and historical references, always in relation to the place).

Table 2 shows a classification of the identified problematics along the Maresme region, organized according to these three research lines. This is the first step to recognize the needs of the territory and to set a complete image to ease the identification of the most representative case studies to be developed (Table 2).

### 3.3. Setting of Action Variables

Once the problem typologies were set, a series of variables could be defined to clarify the landscape conditions of the site. Based on a general overview, these variables were related to the transversal section of the coastline and the landscape elements which were part of it: topography, hydrography, constructions or activities. A combination of these variables and problematics would establish the selection of a varied and valid sample of case studies, which could be developed by means of an urban and architectural project.

Four variables were defined in relation to the coastline conditions (Table 3):

- Variable 1: the typology of occupations in the inner part of the infrastructures in relation to urban settlements and rural areas, considering activities and constructions;
- Variable 2: uses and landscape typologies in the outer part of the infrastructures in relation to the coastline and activities related with the sea, leisure and landscape;
- Variable 3: the topographic difference between both sides of the infrastructures, which is variable along the coastline and defines the conditions for the connection between villages and the sea;
- Variable 4: the distance from the sea and the first line occupied on the inner side of the infrastructures, which is variable along the coastline and defines the relation with the urban habitat from villages toward the sea.

## 4. Results

### 4.1. Urban Regeneration Projects

From the territorial analysis and fieldwork focusing on the three research lines, some representative case studies were selected as a sample to provide solutions to the identified typological problematics and action variables. Each one of these locations was developed by means of an urban regeneration project. A comparison of the solutions provided would allow for establishing a series of general guidelines to be applied to the whole region. Working with particular locations eases the development of real projects, following a methodology from the most general to the most particular and reverting again to a larger scale with the standard strategies.

**Figure 8:**
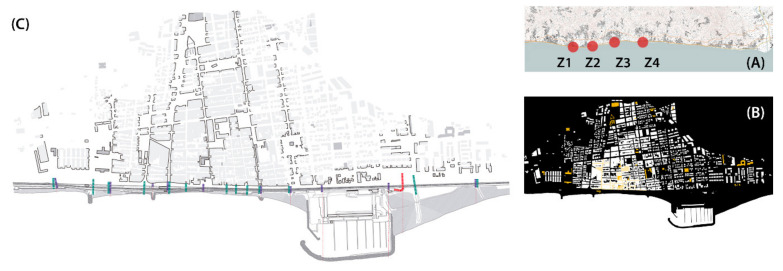
Graphics of the first approaches from urban regeneration projects (Source: authors’ own). (**A**) Location of case studies in the Maresme region. (**B**) Figure and ground diagram of Premià de Mar, with urban structure, public spaces and buildings related to the coastline. (**C**) Transversal street and underground passage catalogue of Premià de Mar.

The proposed solutions have an abstract character to them, as they are based on universal relations between architectonic elements, such as pedestrian walkways, squares, underground passages, courtyards, stairs or ramps. The selection of case studies was based on a combination of typological problematics and action variables, both of which were related to landscape elements, the historical evolution of each site, the potential for urban regeneration and access to required information (Table 4). To develop this phase of the research, four case studies were selected, and the projects are detailed in the following subsections (Figure 8). The descriptions of each case study include some architectural works presented as project references related to materiality, climatic systems or structure.

#### 4.1.1. Downtown of Premià de Mar

This case study was located in the downtown area of Premià de Mar and an example of a linear solution (greenway and civic way) and of public urban spaces connected to crossroads. This village was selected due to its average size in the region, which is accessible for pedestrians and has a compact, historic downtown that sets the maritime façade.

Urban analysis detected the existence of multiple underground passages, most of them being former sewer pipes, which connected streets, squares and facilities to the sea. To give a solution to this aspect, the project focused on a generic section for both a civic way and a greenway, each one located on a different side of the railway. Two public squares appear at the crossroads: one with a more urban character at the end of the downtown street and the other with a more landscaping-based character in the peripheries [70].

The idea of using underground passages to foster accessibility toward the sea was based on several aspects, including sustainable responsibility by reusing the existing passages and improving their design quality and also through landscape integration by aiming to preserve clean views over the zero level and the horizon, which defines the seafront. In addition, these passages need to meet basic functional, comfort and security requirements, considering the relevant risk of flooding.

**Figure 9:**
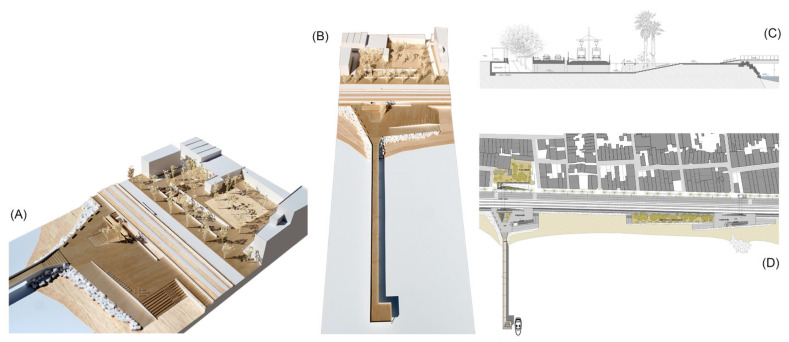
Urban and architectural project for the case study of downtown Premià de Mar (Source: authors’ own). (**A**,**B**) Model pictures of the dock and public squares. (**C**) Transversal section through an underground passage. (**D**) General floorplan with the urban and landscape squares, a lightweight dock and a seaside greenway.

In the littoral square, the project included some facilities related to the beach (e.g., showers, benches and bike rentals). There was also an opportunity to consider the construction of lightweight docks to protect the beaches from sea storms and also provide a new public transport system of boats similar to Venetian vapporetos to diminish the traffic of land infrastructures [71]. Constructive and architectonic criteria applied to solve sections of longitudinal pathways and public squares need to be replicable in the whole littoral [72]. These need to be flexible systems that can be adapted to the diverse conditions of each place and made of industrialized and easily maintainable materials (Figure 9).

#### 4.1.2. Stop on the Beach of Vilassar-Cabrera

The case study of a stop on the beach of Vilassar-Cabrera is an example of parking lodgments along the seafront related to stations and other facilities. This is a common typology of railway stations in the Maresme region. It is located on the outer part of the railway, so the solution is easily repeatable in other stations. Urban voids and uses of parking and public services were considered in this project [73].

Urban analysis showed that this is an inter-urban station with a high influx of users that need to access it by car. On the sand dunes, there is a parking lodgment which can only be reached through an underground passage. Citizens, tourists and patrons of restaurants and other leisure activities also park in this area. The seafront has a discontinuous skyline, with several buildings located in different alignments and a lack of urban and architectural ambience. Furthermore, the pedestrian underground passages are too narrow and distributed irregularly.

The seafront can be solved in a continuous way all along the outer part of the railway. This axis is oriented toward pedestrians, bicycles and service vehicles. All diverse beach establishments are designed with a system of prefabricated constructions to unify the maritime façade [74]. These lightweight constructions are distributed in contact with the seafront to help organize parking lodgments, commercial activities and the platforms of the stop.

**Figure 10:**
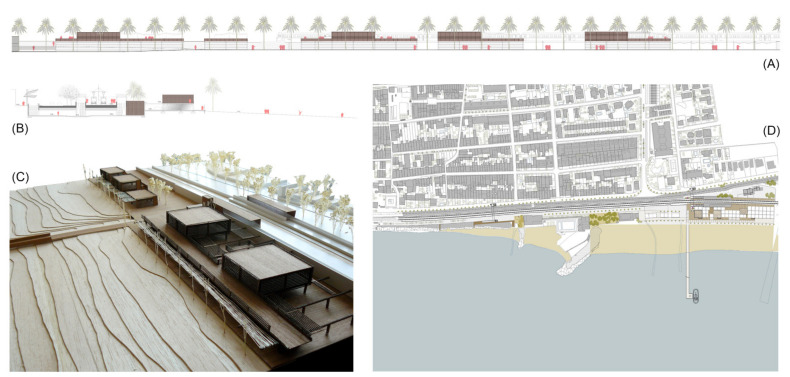
Urban and architectural project for the case study of a stop on the beach of Vilassar-Cabrera (Source: authors’ own). (**A**) General elevation of the maritime façade. (**B**) Transversal section through the underground passage. (**C**) Model picture of the prefabricated constructions. (**D**) General floorplan with different elements used to add order to the maritime seafront: prefabricated buildings, greenway and urban public spaces.

In this case, a construction system solved both facades and roofs at the same level as the railway lines, with attractive open views toward the landscape. These were lightweight and prefabricated constructions made of timber structures and lattices. Due to the usual overlapping with the maritime public domain boundaries, these buildings needed to be temporary and easy to dismantle [75]. Finally, the underground access paths to the platforms were widened and illuminated through patios, following the same criteria of the previous case study [76]. The underground passage for vehicles was also transformed with a more urban treatment related to the adjacent public square, making it adequate for pedestrians (Figure 10).

#### 4.1.3. Railway Station of Mataró

The case study of the railway station of Mataró is an important example, as it is located in the largest town in the Maresme region. This focused on the regeneration of an obsolete historic building to enhance connectivity and to act as a creator of civic activity in the city [77].

Urban analysis detected that this is a traditional, disused building; however, it is located in a strategic position: in the fringe between an urban settlement and the sea. Access to platforms and to the seaside is produced by underground passages with no spatial or comfort qualities. Most of them are former sewer pipes.

The project proposes to widen the existing underground passages and to connect all of them with the basement of the railway station [78]. With this solution, all of these elements can be unified, creating a huge underground lobby with courtyards and vegetation. As such, all the underground connections (e.g., the nautical club, beach, platforms and station) are supported by one unique space that is bigger and better controlled in terms of maintenance and security. This space is not an underground passage anymore but a building with natural light, ventilation and views of the outside [79].

Regarding the existing building of the railway station, it can be refurbished and recovered as a public facility for the city. The ground floor opens to the civic way, and the roof is also accessible to the public, with attractive views over the sea. The intermediary levels can be transformed for public or private use for the third sector (i.e., services, associations, coworkers and start-ups).

**Figure 11:**
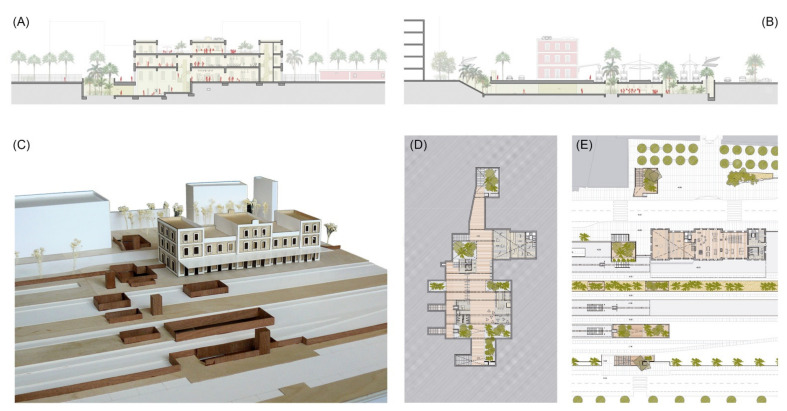
Urban and architectural project for the case study of the railway station of Mataró (Source: authors’ own). (**A**) Section of the historic building and lobby. (**B**) Transversal section of the underground passage. (**C**) Model picture of the station. (**D**,**E**) Basement and ground floor plans with green courtyards and an underground structure following the railway platforms.

This project is thought to be a representative case of obsolete urban buildings. Their integral architectonic regeneration, with the addition of new public uses, transforms these spots into new centralities and nodes of urban activity. In addition, this typology also solves the longitudinal continuity and transversal permeability issues in a unique and clear type of intervention (Figure 11).

#### 4.1.4. Stream Mouth in Llavaneres

The case study of the stream mouth in Llavaneres is an example of crossing between a longitudinal greenway and the ending of an important hydrographic element used as a public space while it is not flooded. The objective was to generate a set of pathways to enhance the transversal mobility inland but in a healthier and more sustainable way [80]. At the same time, as this stream is a public space, eventual floods and the effects on existing constructions were considered (e.g., bridges, restaurants, stations, parking lodgments and facilities).

Urban and landscape analysis detected that this type of stream usually had little water, so it could be regularly used as a public space [81]. However, there are sometimes floods that fill the stream and convert it into a river. The mouth of the Llavaneres stream is a paradigmatic case with its width (about 40 m) and the two bridges that provide continuity of the railway and the N-II road, which solve several topographic unevenness issues. Inside this hydrographic element, there are some spontaneous parking lodgments, and on the beach, there are several disorganized facilities and establishments.

This project aimed to treat all these crossing spaces with an urban character, solving the connection between the longitudinal greenway and the transversal streams all along the coast [82]. Public urban spaces created around the stream mouth and close to the beach can be designed as public squares with strong materials that can support eventual floods [83]. The stream, due to its dimensions and importance, is projected as a green corridor extending to the coastline that is designed using nature-based systems [84].

**Figure 12:**
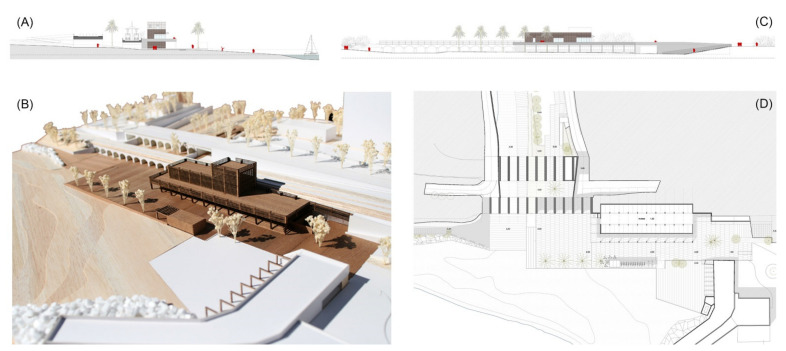
Urban and architectural project for the case study of the stream mouth in Llavaneres (Source: authors’ own). (**A**) Transversal section of the underground passages. (**B**) Model picture of prefabricated constructions. (**C**) General el-evation of the maritime façade. (**D**) General floorplan with green treatment of the stream, floodable squares and light-weight constructions for local services and the railway station.

All the parking lodgments and facilities related to the beach were organized by means of lightweight constructions, using the same prefabricated wooden system in the previous case studies. Due to topographic unevenness, this project included a footbridge over the railway to ease accessibility to platforms and to the beach. Additionally, the public square was equipped with facilities related to sports and healthy activities (e.g., bike rentals, showers and urban furniture) and to renewable energy production (e.g., solar panels and wind turbines) (Figure 12).

### 4.2. Intervention Strategies

Specific resolution of the four urban and architectural projects, understood as typological case studies, would make it possible to visualize diverse situations in a common graphic register (e.g., drawings, 3D models and physical models). Comparison of these projects establishes a compound of action guidelines. These are intervention strategies which can be applied to similar locations along the Maresme littoral or along other coastal regions following the same research methods. These strategies are classified following the three research lines which structure the whole methodology.

#### 4.2.1. Longitudinal Continuity Strategies

These solutions are focused on transforming the N-II road in a civic way and ensuring continuity of the seaside greenway:

- Reduce the road width, with one lane in each direction and an auxiliary third one;
- Widen and urbanize the urban sidewalks to ease the introduction of large courtyards for better access to underground passages;
- Widen the sidewalk between the N-II road and the railway by getting rid of the concrete protections (New Jerseys), and small courtyards and staircases can provide light and access to underground passages;
- Ensure continuity for pedestrians and bicycles by means of a two-way greenway along the seaside;
- Connect the seafront with the inner side of the infrastructures, with a regular distribution of underground passages at a local scale and green corridors through the main streams at a regional scale;
- Transform railway protections at the seaside (i.e., in contact with the longitudinal greenway) using stone gabions and vegetation;
- Foster longitudinal light mobility with a regular distribution of facilities related to bicycles, beach services and renewable energy generation;
- Promote alternative public transport systems such as bike rentals or ferries between Maresme villages and Barcelona;
- Construction of lightweight docks to protect beaches from sea storms, provide service to ferries and show a new sight of the coast as a leisure and tourism landmark.

#### 4.2.2. Transversal Permeability Strategies

These solutions are focused on fostering accessibility to the sea from the urban settlements located further inland:

- Transform existing underground passages instead of building new ones;
- Use the most suitable existing passages in relation to flood prevention (i.e., those that are not directly facing a street toward the sea);
- Provide universal accessibility to all underground passages by means of staircases, ramps and elevators;
- Prevent the direct entrance of rainwater by means of massive handrails and topographical modification of access routes to underground passages;
- Improve the ambience of these passages to make them more secure and comfortable by widening them and adding natural light and ventilation, and as the N-II road is reduced, the passages’ lengths will decrease equally;
- Enlarge access paths to underground passages, with two on both sides and one in the middle;
- Use vertical elements in the access points, such as elevators or wind turbines, to create a recognizable landmark repeated regularly along the coast;
- Add regular footbridges related to light constructions to solve high unevenness;
- Offer new uses for the basements of public buildings related to underground passages, such as adding courtyards and vegetation (e.g., station lobbies and access to platforms);
- Modify underground passages for vehicles to move from motorway aesthetics toward a more urban one, with pavement and furniture such as those in public spaces.

#### 4.2.3. Urban Landscape Strategies

These solutions are focused on providing a unified image to the littoral landscape that is easy to understand from both sides (inland and sea):

- Add new activities to obsolete buildings, parking lodgments and huge commercial complexes in the seafront to highlight them as catalysts for urban regeneration;
- Recognize historic railway stations as part of the collective memory of each place and connect their basements to underground passages, creating activity nodes;
- Open the ground floors of public buildings toward adjacent urban spaces to enhance recognition and active use of these facilities;
- Organize facilities, parking lodgments, stations, commercial areas and beach services by means of lightweight prefabricated constructions that are easy to dismantle;
- Rethink the urban character of nautical clubs and marinas to enhance the continuity of seaside greenways as landmark elements;
- Transform the boundaries of private enclosures (such as campsites, sports fields and nautical clubs) into wide edges with vegetation related to the littoral landscape.

## 5. Discussion

Once the architectural strategies are defined and classified in each of the three research lines, the last step of the methodology consists of defining a matrix of standardized design solutions. This document is a diagnosis tool to be used during the brainstorming stage of urban and architectural projects developed to solve the problematics detected along Maresme’s littoral. Each one of these proposed solutions was related to a category regarding its effects on mobility and landscape: 1_Longitudinal, 2_Transversal, 3_Crossing and 4_Building.

This first stage of research concluded with a definition of eight standard architectural solutions organized by pairs in each category. As a catalogue, these strategies could be applied along the territory with the aim to solve the multiple combinations between typological problematics and action variables, both of which were identified at the beginning of this research.

**Figure 13:**
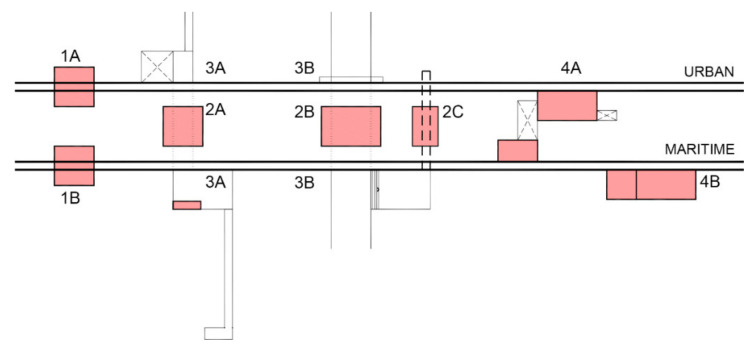
Matrix of standardized design solutions (Source: authors’ own).

The matrix of standardized design solutions is graphically defined in Figure 13 as a scheme which shows the two infrastructure sides (urban above and maritime below) and different types of transversal passages. The conceptual schemes of the eight standardized solutions are drawn in red. In addition, each one of the architectonic strategies is represented with a generic virtual model (3D views, floorplans and sections), which provides the recommended dimensions and materials to be applied in forthcoming architectural projects promoted by local administrations (Figure 13).

**Figure 14:**
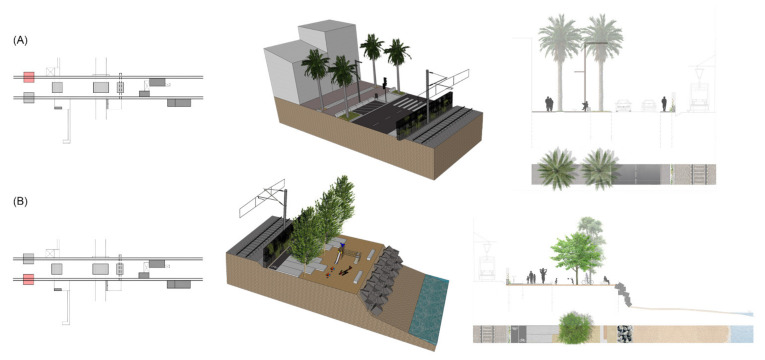
Standardized solutions for longitudinal typologies (Source: authors’ own). (**A**) Urban side and civic way. (**B**) Maritime side and greenway.

1A_ Longitudinal/Urban represents the typological solution for the urban civic way. The urban sidewalk is widened with pavement and trees. The N-II road is reduced, with two lanes for circulation and a third auxiliary one for bus stops, parking and stop and go. Protections with the railway are kept. This is the basic typological section which is extensible to the whole littoral (Figure 14A).

1B_Longitudinal/Maritime represents the typological solution for the greenway along the seaside. It consists of a two-way pathway for both pedestrians and bicycles. When the topography is reduced or too steep, the solution provides concrete prefabricated panels which can be fixed, cantilevered or solved as a bench with a handrail. In those sections with more terrain, the solution includes vegetation, urban furniture and facilities. This is also an extensible solution for the whole littoral (Figure 14B).

**Figure 15:**
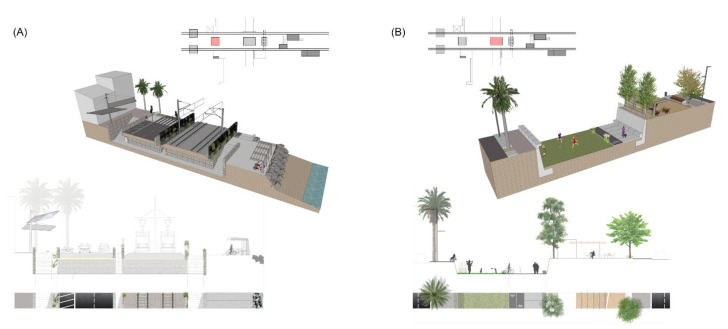
Standardized solutions for transversal typologies (Source: authors’ own). (**A**) Underground public space, non-floodable. (**B**) Underground public space, floodable.

2A_Transversal/Underground/Non-floodable is the definition of a typology of passage understood as the transformation of an existing one. It contains three access courtyards: two in the extremes and one in the center. Passages are accessible by means of elevators, ramps and staircases located following a longitudinal circulation. Canopies for water protection and wind turbines are located close to access points to be recognized as landmarks. Flood prevention is taken into account by their location far from the main streams and also by including solid handrails and podiums. Interior wall treatment of the tunnels is proposed with light-colored ceramics that are easy to maintain (Figure 15A).

2B_Transversal/Underground/Floodable is a solution that focuses on the design of streams with eventual water flow but of a high intensity. These are planned to be green connections for pedestrians and bicycles toward the interior of the region. The riverbank edges are designed with concrete walls and vegetal treatment or also with steps for leisure activities. The access points are based on ramps and staircases linked to sidewalks at the street level, where prefabricated constructions can be distributed with restaurants and other facilities protected from water. The riverbank is treated with autochthonous Mediterranean vegetation, as are the upper sidewalks (Figure 15B).

**Figure 16:**
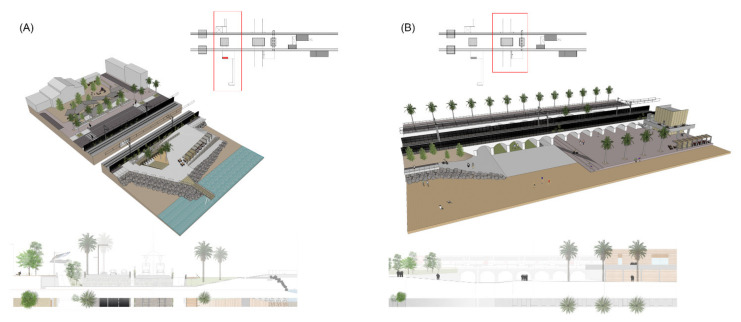
Standardized solutions for crossing typologies (Source: authors’ own). (**A**) Non-floodable crossing, both urban and maritime. (**B**) Floodable crossing, both urban and maritime.

3A_Crossing/Non-floodable/Urban and Maritime is a typology that solves the crossing of longitudinal and transversal axes, which can be combined along the littoral. The solution provides two types of squares in relation to their location: urban or maritime. These public spaces are wide and have clear geometries. Materiality is defined with soft and hard pavements, vegetation, urban furniture and facilities for bikes, ferries and beach services. Access to underground passages is solved with large courtyards which can be covered with canopies. Urban squares, usually located downtown, are connected to the docks (Figure 16A). 

3B_Crossing/Floodable/Urban and Maritime is a typology that solves the crossing of longitudinal greenways and transversal streams. These are nodes for pedestrians and bicycles but also access points for service vehicles (e.g., for maintenance and restaurant supplies at the beach level). The solution considers the real possibility of having this urban space flooded. For this reason, the topography is adapted with paved platforms with concrete prefabricated panels and vegetation along the edges. Wooden modular constructions can be distributed to add order to existing commercial activities and parking lodgments (Figure 16B).

**Figure 17:**
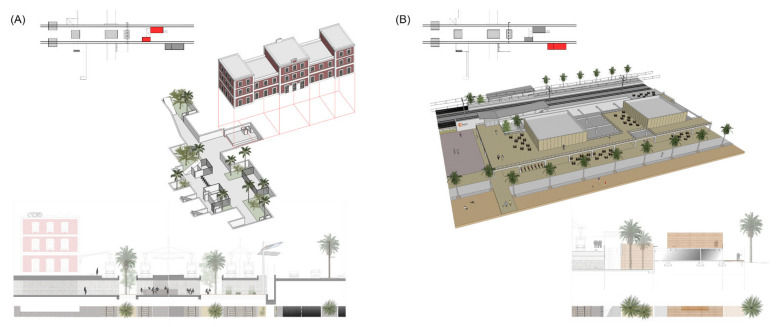
Standardized solutions for building typologies (Source: authors’ own). (**A**) Urban side, civic way. (**B**) Maritime side, greenway.

4A_Building/Urban is a typology focused on the transformation of existing buildings, with the addition of new uses and the creation of a connection between longitudinal and transversal mobilities. Buildings are developed in the basement, sharing spaces with existing underground passages for access to railway platforms or to the beach. These spaces are understood as lobbies with courtyards, skylights and vegetation. The rest of the building is refurbished to support public uses. The ground floor can be opened toward the civic way and the city. This solution is mainly for obsolete railway stations, but it can be applied to other buildings, such as factories or facilities (Figure 17A).

4B_Building/Maritime is a typology focused on defining a modular system to build dismountable constructions in order to unify the urban landscape along the longitudinal greenway. This system can organize all different kinds of uses and activities on the coastline, including restaurants, commercial businesses, nautical clubs, beach services, railway stops and parking lodgments. In areas of high unevenness, isolated elements can be hidden with canopies that add new covered areas to host outdoor activities. These constructions are prefabricated, being made of timber structures and lattices, fabrics and vegetation (Figure 17B).

## 6. Conclusions

This research provides a method to obtain standard solutions in order to solve coastline mobility conflicts produced by linear infrastructures. This process occurs in three dimensions: (1) ensuring longitudinal continuity by means of a greenway all along the seaside, (2) reinforcing transversal permeability between the inland areas and the seaside by means of a sequence of connections beneath the linear infrastructures and (3) unifying the qualities of the littoral urban landscape by organizing its maritime façade, its public spaces and its buildings and supporting social activities.

Application of these guidelines will result in a pleasant commuting route for inhabitants and tourists to be used not only for daily local trips or toward Barcelona, but also during weekends and holidays. In fact, many administrations in the county are focused on these ideas to regenerate the coastline in a more civic way. For example, the Mataró municipality is currently developing a proposal for a new underground station [85]. Furthermore, the Catalan government has promoted free circulation through inland highways, which has great potential for unloading the traffic on the N-II road in order to convert it into a civic way [86].

A relevant aspect of this research was to introduce the architectonic project practice into research methods. The four representative proposals ease the identification and further application of generic urban design elements, such as civic roads, greenways, urban and maritime squares, underground passages or modular structures. Due to their standard nature, these solutions can be combined to solve conflicts in all different locations along the studied territory. The matrix of standardized design solutions is a tool to identify the starting point for each specific urban project under a homogeneous criterion for the whole region. Consequently, the most suitable solutions for each site need to be adapted in relation to the contextual conditions by means of a professional architectural project. 

In conclusion, this research provides a method to define the basic system of solutions and also the process to combine them so as to be easily replicable. Considering the Maresme region as a case study, the urban or architectural design that will follow the research will regenerate the coastal public spaces, converting them into urban references all located at the crossings between transversal and longitudinal ways. In a wider scope, the intention of this research was to assist administrations with solving local problematics but with common criteria. Therefore, there is no need for a large-scale urban project, which is usually expensive and complex. On the contrary, the intervention strategies proposed are economically sustainable and affordable for a wide range of administrations and stakeholders, and they are also flexible enough to be developed through varied deadlines set by different municipalities.

This research provides a common base of discussion defined by comprehensible tools to explain and develop urban projects. With the idea of solving the issues of a territory as a whole by means of small interventions, all the solutions provided are coherent and organized in a common design tool. This overview establishes general design bases to organize the whole maritime façade of the Maresme region in relation to its particular conflicts while taking into account its landscape values. However, while the standard solutions identified might be coherent for Maresme, they may not be suitable for other Mediterranean territories. The value of this research is in its capacity to be repeated in other regions by following the process and methods to set a customized matrix for each location which is closely related to its specific conflicts and its landscape conditions.

In order to answer the three aforementioned research questions (see Section 2), this study established a specific methodology. First, the littoral fringe was analyzed in a homogeneous way by registering and classifying the problematics both virtually and with repeated fieldwork visits. In addition, intangible aspects were taken into account to understand the current problematics, such as the historic, social or cultural characteristics of the region. Second, in-depth analysis of the specific site was required prior to selection and proposal of the case study projects to achieve universal solutions. In this phase, basic design tool decisions were important to finally come to adequate but generic solutions, such as work scale, materiality or modeling parameters. Finally, the last phase involved synthesis of the solutions by reducing the design proposals to a set of essential elements, dimensions and materials.

Throughout this process, the use of graphic tools is fundamental, and decisions are taken in terms of the research methods from the analysis (e.g., digital cartographies, schematic mapping and handmade sketches) to the design (e.g., models, site plans and 3D images) and, finally, the matrix of solutions (e.g., standard sections, cuts and 3D sketches). By means of graphic aspects, this research establishes tools to register, compare, decide, propose and show a proposal through an intelligible code for both citizens and administrations.

The next step in this research aimed to register all the problematics and variables identified in a general map of the region by means of Geographical Information Systems (GISs) [87]. This forthcoming document will include georeferenced locations related to the characteristics of each place. Once the locations were properly identified in the map, the matrix of standardized design solutions would add new data regarding which intervention strategies could be applied in each place. This process would set the basis for further architectonic projects promoted by different municipalities (Table 5). To provide the maximum potential to this second phase of the research, this GIS mapping tool should be designed with a clear layout and be linked with official cartographies in order to foster its use by administrations at different scales and also by public and private stakeholders.

## Figures and Tables

**Table 1:** Research process based on IAR’s “Mediterranean Strategies” methodology.

Methodology	Analytical Stage	Proposal Stage
**Analysis**	(1) Analysis of the territory, history, culture, economy and traditions	(2) Identification and classification of typological problematics
**Regeneration projects**	(3) Selection of representative case studies and context analysis	(4) Proposal of transformation, specific models and solutions and typologies
**Intervention strategies**	(5) Four types of solutions: site, project, construction and climate	(6) Definition of action guidelines for unitarian territorial regeneration

**Table 2:** Identification of typological conflicts classified by research lines (Source: authors’ own).

Longitudinal Continuity	Transversal Permeability	Urban Landscape
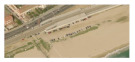 L1_Parking lodgments	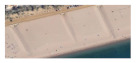 T1_Beach stream mouths	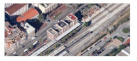 P1_Railway stations
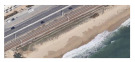 L2_Inadequate pavement	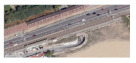 T2_Vehicle underground passage	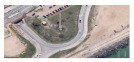 P2_Abandoned factories
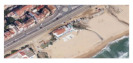 L3_Seafront not urbanized	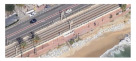 T3_Pedestrian underground ways	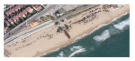 P3_Parking lodgments
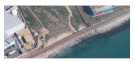 L4_Streams	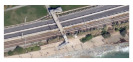 T4_Elevated footbridges	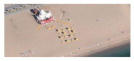 P4_Temporary beach buildings
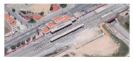 L5_Railway stations	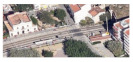 T5_Railroad crossing	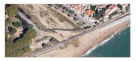 P5_Nautical clubs and facilities
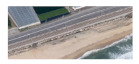 L6_Broken pathways	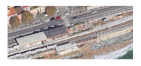 T6_Station underground passages	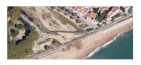 P6_Footbridges
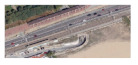 L7_Vehicle underground passage	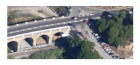 T7_ Pedestrian private passages	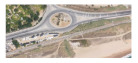 P7_Roundabouts
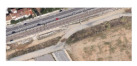 L8_End of pathway	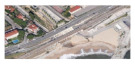 T8_Natural streams	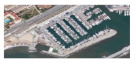 P8_Marinas
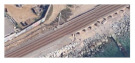 L9_Topographic change	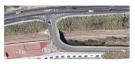 T9_Elevated vehicle passages	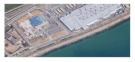 P9_Factories and commercial
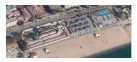 L10_Seafront facilities	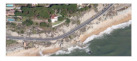 T10_Natural footbridges	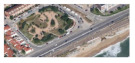 P10_Abandoned urban voids
		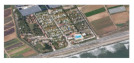 P11_Campsites

**Table 3:** Classification of the action variables and landscape elements (Source: authors’ own).

Variable 1		Variable 2	
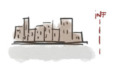 V1A_Large urban consolidated	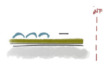 V1F_Crops	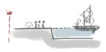 V2A_Marina	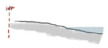 V2F_Beach
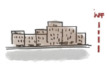 V1B_Small urban consolidated	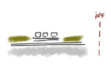 V1G_Transport	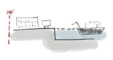 V2B_Fish harbor	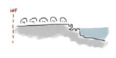 V2G_Parking/Rocks
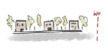 V1C_Isolated urbanconsolidated	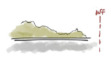 V1H_Leisure nature	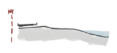 V2C_Seafront/Beach	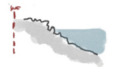 V2H_Rocks
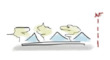 V1D_Urban campsite		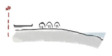 V2D_Circulations/Parking/Beach	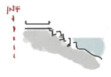 V2I_Seafront/Rocks
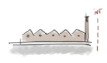 V1E_Industry		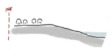 V2E_Parking/Beach	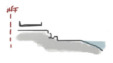 V2J_Seafront/Rock/Beach
**Variable 3**	Topographic difference between both sides		
**Variable 4**	Distance between inhabited urban front and the beach		

**Table 4:** Case study selection with identification of problematics, research lines and variables.

Case Study	Problematics ^1^	Variables
Downtown of Premià de Mar	L3 T1 + T3 P4 + P10	V1H + V2C + V3 + V4
Stop on the beach of Vilassar-Cabrera	L1 + L3 + L7 T1 + T2 + T3 + T6 P3 + P4 + P5	V1A + V2D + V3 + V4
Railway station of Mataró	L1 + L5 + L10 T3 + T5 + T6 P1 + P10	V1B + V2A + V3 + V4
Stream mouth in Llavaneres	L3 + L4 + L7 + L10 T8 + T3 + T2 + T1 P4 + P5 + P10	V1H + V2C + V3 + V4

^1^ Longitudinal continuity (L), transversal permeability (T) and urban landscape (P).

**Table 5:** Standardized solutions applied to case studies.

Case Studies	Standardized Solutions
Downtown of Premià de Mar	1A + 2A + 3A
Stop on the beach of Vilassar-Cabrera	1A + 2A + 4B
Railway station of Mataró	1A + 2A + 4A
Stream mouth in Llavaneres	1A + 2B + 3B + 3C

## Data Availability

Not applicable.
